# Targeting TLR4 alleviates the progression of experimental autoimmune encephalomyelitis by regulating central resident cells and Th1/Th17 cell differentiation

**DOI:** 10.3389/fimmu.2026.1973420

**Published:** 2026-10-16

**Authors:** E Du, Ke Lei, Wei Deng, Siyu Yang, Yun Liu, Jiapei Dai, Jiawen Lei, Rui Zhang, Hao Tao, Huoying Chen, Yan Sun

**Affiliations:** 1 College of Life Sciences, South-Central Minzu University, Wuhan, Hubei, China; 2 Wuhan Institute for Neuroscience and Neuroengineering, South-Central Minzu University, Wuhan, Hubei, China; 3 State Key Laboratory of Virology, Wuhan Institute of Virology, Chinese Academy of Sciences, Wuhan, China; 4 Department of Laboratory Medicine, The Second Affiliated Hospital of Guilin Medical University, Guilin, China; 5 School of Undergraduate Studies, South-Central Minzu University, Wuhan, Hubei, China; 6 Guangxi Key Laboratory of Metabolic Reprogramming and Intelligent Medical Engineering for Chronic Diseases, Guangxi Key Laboratory of Multimodal Biomarkers and Precision Diagnosis, Guilin Medical University, Guilin, China

**Keywords:** experimental autoimmune encephalomyelitis, HMGB1, neuroinflammation, Th1, Th17, toll-like receptor 4 (TLR4)

## Abstract

Multiple sclerosis (MS) is an autoimmune demyelinating disease characterized by neuroinflammation. Toll-like receptor 4 (TLR4) is a receptor for damage-associated molecular patterns, such as high-mobility group box 1 (HMGB1) and plays a role in innate immunity. However, its precise role and cellular dynamics in MS pathogenesis are unclear. Using the experimental autoimmune encephalomyelitis (EAE) model, we mapped the expression dynamics of TLR4 and found that in the spinal cords, the cell type associated with the disease shifted from neurons to activated glia. To establish causality, we subjected *TLR4*^-^/^-^ mice to EAE and observed significantly attenuated disease severity, reduced demyelination, and preserved neuronal integrity, alongside suppressed microglial and astrocytic activation. Furthermore, TLR4 deficiency was not merely dampening CNS-localized inflammation but was also associated with suppressed peripheral MOG_35–55_-specific Th1/Th17 effector responses, as evidenced by reduced production of IFN-γ and IL-17A in lymphoid organs, together with reduced TNF-α, as well as diminished expression of the Th1/Th17 lineage-defining transcription factors T-bet and RORγt in the spinal cord infiltrates. This suggests that TLR4 plays a key role in EAE pathology by connecting peripheral Th1/Th17 differentiation with central glial inflammatory responses. Critically, to translate these findings, we evaluated the small-molecule TLR4 antagonist TAK-242. While prophylactic administration was effective, therapeutic dosing initiated after disease onset still significantly alleviated clinical scores and delayed progression, highlighting its potential for clinical intervention. Overall, our results demonstrate that targeting TLR4 simultaneously curtails pathogenic T-cell differentiation and local neuroinflammation, establishing TLR4 as a dual-action therapeutic target for MS.

## Highlights

- TLR4 expression is upregulated in EAE spinal cord with a neuron-to-glia shift.
- TLR4 deficiency alleviates EAE clinical severity and neuropathology.
- TLR4 knockout suppresses peripheral and central Th1/Th17 differentiation.
- Treatment with TAK-242 treatment confers prophylactic and therapeutic effects against EAE.

## 1 Introduction

Multiple sclerosis (MS) is a chronic inflammatory demyelinating disorder of the central nervous system (CNS) that affects over 2.3 million people worldwide and imposing a significant socioeconomic burden (1). Although current immunomodulatory therapies, such as interferon (IFN)-β, corticosteroids, fingolimod and monoclonal antibodies (mAbs) can reduce relapse rates and improve quality of life, they are frequently accompanied by serious adverse effects (2). The pathological hallmarks of MS include inflammation, gliosis, axonal degeneration and loss of the myelin sheath in the brain and spinal cord, which leads to progressive neurological disability. A key initiating event is the breakdown of the blood-brain barrier (BBB), allowing peripheral immune cells such as myelin-reactive T lymphocytes, macrophages, dendritic cells and neutrophils to infiltrate the CNS and fuel a self-perpetuating neuroinflammatory cascade (3). This process is recapitulated in the primary experimental model of MS, experimental autoimmune encephalomyelitis (EAE). Given the limitations of existing therapies, identifying novel molecular targets that critically drive this neuroinflammatory axis is a pressing challenge.

High-mobility group box 1 (HMGB1) is a nuclear protein that functions as a damage-associated molecular pattern (DAMP) and has emerged as a promising therapeutic target implicated in the pathogenesis of EAE/MS. Under inflammatory conditions, HMGB1 is actively secreted by stimulated immune and non-immune cells, or passively released from damaged or necrotic neurons and glia. Extracellular HMGB1 functions as a potent pro-inflammatory cytokine by signaling through receptors including Toll-like receptor (TLR2), TLR4, and the receptor for advanced glycation end products (RAGE), which are expressed on macrophages, microglia and neurons (4). This signaling induces the production of pro-inflammatory cytokines (e.g., TNF-α, IL-1β, IL-6) and chemokines, amplifying immune cell recruitment and activation. Notably, a pathogenic feedback loop is established, as proinflammatory cytokines such as TNF-α can further stimulate HMGB1 translocation into the cytoplasm and release from neurons (5). Elevated levels of HMGB1 in the serum, cerebrospinal fluid (CSF) and spinal cord homogenate strongly correlate with EAE severity across disease stages (pre-onset, onset, peak, and remission). Preclinically, interventions targeting HMGB1, such as neutralizing monoclonal antibodies, have been shown to suppress EAE severity. Intracerebroventricular injection of anti-HMGB1 mAbs clearly suppresses EAE progression (6). Pharmacological inhibition of HMGB1 using glycyrrhizin (a natural HMGB1 inhibitor) reduces HMGB1 expression and TNF-α-mediated HMGB1 release from neuronal cells, thereby exerting anti-inflammatory properties and providing neuroprotection against EAE (5, 7). These findings highlight the pivotal role of HMGB1 in neuroinflammation and its potential as a therapeutic target for MS/EAE.

Neuroinflammation contributes to the progression of many neurodegenerative diseases. Toll-like receptors (TLRs) play an important role in the development of chronic inflammation in these conditions. TLR4, the primary receptor for HMGB1, plays a key role in regulating innate immune activation and inflammation. TLR4 has also been associated with various neurodegenerative diseases, including Parkinson’s disease (PD) (8), Alzheimer’s disease (AD) (9) and Huntington’s disease (HD) (10). Notably, elevated levels of HMGB1 and soluble TLR4 in the CSF and serum of MS patients correlate with disease activity and prognosis. Recent studies have found that TLR4 mediates Th17 cell infiltration via the CCL25/CCR9 signaling pathway during EAE pathogenesis (11). *In vivo*, treatment with LPS or other TLR4 ligands exacerbates EAE, whereas targeted disruption of TLR4 prevents disease development (12). Taken together, these data support a model in which HMGB1, which is released during neuronal damage and demyelination, activates TLR4 signaling, thereby contributing significantly to MS/EAE neuroinflammation.

TAK242 (also known as Resatorvid or CLI-095) is an exogenous, synthetic, small-molecule antagonist of TLR4. It has been shown to cross the blood-brain barrier, exhibit neuroprotective effects in models of cerebral ischemia, and bind to the TIR domain of the receptor, disrupting the interaction between TLR4 and its adaptor molecules, thereby inhibiting downstream signaling events (13). TAK242 offers a potential strategy for dampen MS/EAE-associated neuroinflammation in a targeted manner, potentially without interfering with relevant adaptive immunity or other TLR-mediated innate responses. However, the precise mechanisms and full therapeutic potential of TLR4 inhibition specifically within in the context of EAE-driven neuroinflammation require further elucidation.

Therefore, this study aimed to investigate the expression, cellular distribution, and functional role of TLR4 in the spinal cord during EAE progression. We further hypothesized that pharmacological inhibition of the HMGB1/TLR4 axis using TAK-242 would attenuate EAE severity by modulating pathogenic Th1/Th17 responses and protecting neuronal integrity, thereby validating TLR4 as a promising and druggable target for neuroinflammatory disorders such as MS.

## 2 Materials and methods

### 2.1 Mice

Specific pathogen-free (SPF) female C57BL/6J mice, aged 7-8 weeks were obtained from Beijing Vital River Laboratory Animal Technology Co., Ltd. (Beijing, China), and were randomly assigned to different experimental groups. TLR4-deficient mice (carrying a deletion of the TLR4 gene, [*TLR4*^−/−^] on a C57BL/10ScNJNju background) were purchased from the Model Animal Research Centre of Nanjing University (Nanjing, China) and bred for 12 generations onto the C57BL/6J background. All mice were maintained under pathogen-free conditions (at 22-25 °C with a 12-hour light-dark cycle) and were given standard laboratory chow and water ad libitum. The animals were kept under special sterile conditions at the South-Central Minzu University Institutional Animal Care and Use Committee, and all parameters were normal.

### 2.2 EAE induction and assessment

Female C57BL/6J mice and *TLR4*^-/-^ mice were immunized with subcutaneous (s.c.) injections of a 2 mg/mL solution of the myelin oligodendrocyte glycoprotein (MOG_35–55_) peptide (MEVGWRSPFSRVVHLYRNGK; ChinaPeptides Co., Ltd., Shanghai, China) in equal volumes of 0.1 M phosphate-buffered saline (PBS, pH 7.6) and complete Freund’s adjuvant (Cat#F5881, Sigma-Aldrich, St. Louis, MO, USA), containing 5 mg/mL of heat-killed *Mycobacterium tuberculosis* H37Ra (Cat#231141, Difco Laboratories, Detroit, MI, USA). Then, 250 ng of pertussis toxin (Cat#2980, Sigma-Aldrich) was administered intraperitoneally (i.p.) on the day of the MOG_35–55_ injection and again two days later. The mice were monitored daily, and clinical disability was graded on a scale from 0 to 5 (0, healthy; 0.5, weak tail; 1, limp tail; 2, hind limb weakness; 2.5, one hindlimb paralyzed; 3, both hindlimbs paralyzed; 3.5, forelimb weakness and hindlimb paralysis; 4, tetraplegia; and 5, moribund state or death) as previously described. The progression of EAE was categorized into four stages based on clinical symptoms. The pre-onset stage occurs 5 - 10 days after induction and is characterized by mice showing no clinical symptoms. The onset stage occurs 11 - 14 days post-induction, characterized by mice with a score of 0.5 or 1. The peak stage occurs 17 - 21 days post-induction and is characterized by mice with a score of 3 or 4. The chronic stage occurs after 31 - 36 days and is characterized by a reduction in score from 3 or 4 to 1 or 0.5.

### 2.3 Treatment of EAE mice with TAK-242

The TLR4 antagonist TAK-242 (1-cyclohexene-1-carboxylic acid, 6- [((2-chloro-4- fluorophenyl) amino) sulfonyl] ethyl ester, (6R)) was obtained from MedChemexpress (Cat#HY-11109, Monmouth Junction, NJ, USA). It was dissolved in dimethyl sulfoxide (DMSO; Amresco, Solon, OH, USA) and then diluted in 0.9% saline. The mice were injected intraperitoneally (i.p.) with TAK-242 (25 mg/kg body weight) every other day from days -1 to 29 (strategy I), from days 11 to 21 (strategy II), or from days 15 to 23 (strategy III) after EAE induction, respectively. The dose of TAK-242 used in this study is within the selective window reported previously (14, 15). Animals were monitored daily throughout the whole experiment.

### 2.4 Tissue preparation

Nineteen days after MOG_35–55_-immunization, the mice were sacrificed under deep anesthesia by intracardial perfusion with 0.9% saline (37°C), followed then by 4% paraformaldehyde (PFA; Merck, Darmstadt, Germany) in PBS. The brain and thoracic spinal cord tissues were then removed, post-fixed in 4% PFA at 4 °C for 24 h and drained for 72 h in 30% sucrose in 0.05 M Tris-buffered saline (TBS; 0.05 M Tris, 0.9% NaCl, pH 7.6) at 4°C. The tissues were then cut into 20 μm slices for histopathological, immunohistochemical and immunofluorescent staining.

### 2.5 Histopathological and immunohistochemical analysis

For the histopathological analysis, spinal cord sections measuring 20 μm in thickness were stained with either Luxol Fast Blue (LFB; Sigma-Aldrich) or haematoxylin and eosin (H&E; Sigma-Aldrich). The sections were then observed under a microscope to assess demyelination or visualize leukocyte infiltration, respectively. The assessment of inflammatory cell infiltration in spinal cord sections using LFB staining was performed as previously described. Images were captured using an Eclipse 90i microscope (Nikon, Japan). Demyelination was graded based on LFB staining as follows: 0, no demyelination; 1, rare demyelinated foci; 2, < 25% of the white matter (WM) area; 3, 25%–50% of the WM area; 4, > 50% of the WM area.

For the IHC analysis, the sections were first treated with 0.3% hydrogen peroxide in TBS for 15 min to quench endogenous peroxidase, after which they were washed three times with TBS. The sections were then incubated in 5% bovine serum albumin (BSA) for 2 h at room temperature to block non-specific binding. After incubation with the primary antibody at 4 °C overnight, such as anti-rabbit antibody to Iba1 (1:500; Abcam, ab178846) and NeuN (1:200; Abcam, ab177487), anti-mouse antibody to GFAP (1:1000; Abcam, ab10062), the sections were washed three times with TBS. They were then incubated with an appropriate biotinylated anti-rabbit or anti-mouse immunoglobulin (Ig)G antibody (1:400) for 2 h at room temperature, incubated in a peroxidase-coupled avidin-biotin reagent (1:800; ABC kit, Vector Laboratories, Germany) and visualized using 3,3′-diaminobenzidine (DAB; Dako, Germany), after which they were counterstained with hematoxylin, if necessary. Observation and quantitative assessment of Iba positive (Iba^+^), GFAP positive (GFAP^+^) and NeuN positive (NeuN^+^) cells in the total section, white matter (WM) or grey matter (GM) were performed as previously described. Images were taken using a motorized microscope (ECLIPSE 90i, Nikon, Japan), and quantitative image analysis was performed using ImagePro.

### 2.6 Immunofluorescence double labeling

For IF double labelling analyses, sections were blocked in TBS containing 5% BSA and incubated with the primary antibodies at 4°C overnight. The sections were then incubated with the following primary antibodies for 2 h at room temperature: anti-TLR4 (1:200; Servicebio Technology, Cat#GB11519-50), anti-NeuN (1:200; Abcam, Cat#ab177487), anti-Iba1 (1:500; Abcam, Cat#ab178846), and anti-GFAP (1:1000; Abcam, Cat#ab10062), followed by an overnight incubation (> 14 h) at 4 °C, and an additional 2 h at room temperature. After thorough washing, the sections were incubated with Cy3-, FITC-, Alexa 488-, or Cy5-conjugated secondary antibodies (1:100; CWBIO, Beijing, China) for 1 h at 37 °C. Following three washes using 0.1% Triton X-100 in TBS (TBST), cell nuclei were counterstained using 4’,6-dia2-phenylindole (DAPI; Invitrogen, Carlsbad, CA, USA) for 10 min. The sections were then mounted using an anti-fade medium (Dalian Meilun Biotechnology, MA0221-1-Nov-13J). Images were acquired using a laser scanning confocal microscope (STELLARIS 5, Leica Microsystems, Germany) and analyzed using ImageJ software (Media Cybernetics). The data are presented as the number of positive cells per square millimeter.

### 2.7 RT-qPCR

After perfusion, the spinal cord tissue was flushed out of the spine and physically dissociated using TRIzol reagent (Invitrogen, Carlsbad, USA) to extract the total RNA. cDNA was synthesized using a RevertAid First Strand cDNA Synthesis Kit (Thermo Fisher Scientific, Waltham, USA), after which quantitative PCR with reverse transcription PCR (RT-qPCR) was performed using 2 × SYBR Green Real-Time PCR Master Mix (Absin, Shanghai, China) in a 96-well plate on a QuantStudio 5 instrument. Relative expression levels were estimated using the 2^-ΔΔCt^ method, normalizing to GAPDH expression levels. The primer sequences were synthesized by Sangon Biotech (Shanghai, China) and used as follows:

*Tnf-α* (FW): CCTCCCTCTCATCAGTTCTATGG;

*Tnf-α* (RE): CGTGGGCTACAGGCTTGTC;

*Ifn-γ* (FW): TGGCATAGATGTGGAAGAAAAGAG;

*Ifn-γ* (RE): TGCAGGATTTTCATGTCACCAT;

*Il-17A* (FW): GCTCCAGAAGGCCCTCAGA;

*Il-17A* (RE): AGCTTTCCCTCCGCATTGA;

*T-bet* (FW): CCTCGCACCTGGAGCTGGCTG

*T-bet* (RE): TTATCAGTTGGGAAAATAGTTA

*RORγt* (FW): AGTGTAATGTGGCCTACTCCT

*RORγt (*RE): GCTGCTGTTGCAGTTGTTTCT

*Gapdh* (FW): TGTGTCCGTCGTGGATCTGA;

*Gapdh* (RE): TTGCTGTTGAAGTCGCAGGAG.

### 2.8 Spleen and DLN cell culture

The spleen and DLN were collected, and a single-cell suspension was cultured in 48-well plates at a concentration of 4 × 10^6^ cells/mL per well. The cells were then stimulated with either the culture medium alone or 50 μg/mL of the MOG_35–55_ peptide. After 72 h, the culture medium was collected, and the concentrations of selected cytokines were measured using ELISA.

### 2.9 Preparation of spinal cord homogenate and serum for ELISA

The frozen thoracolumbar spinal cord of each mouse was homogenized on day 19 in phosphate-buffered saline (PBS) containing a protease inhibitor cocktail, then centrifuged at 14,000 × g for 10 minutes at 4 °C. The supernatant was collected, and the concentrations of cytokine were immediately measured using ELISA kits. Blood was collected via the axillary artery, left to clot stand at room temperature for 1 h, and then centrifuged at 3,000 rpm for 15 min at 4 °C to remove the clotted matter and collect the serum. Cytokines concentrations are expressed in pg/g of total protein.

### 2.10 Enzyme-linked immunosorbent assay (ELISA)

The levels of mouse TNF-α, IFN-γ and IL-17A in serum, spinal cord homogenate and culture medium were also assayed by ELISA in according to the kit instructions (ELISA kits: TNF-α, BioLegend, San Diego, CA, USA; IFN-γ and IL-17A, San Diego, CA, USA). Cytokine concentrations were determined using the relevant standard curves.

### 2.11 Statistical analysis

The experimental data were analyzed using GraphPad Prism 10.0 (GraphPad Software, Inc., San Diego, California, USA). The data are presented as the mean ± standard error of the mean (SEM). Comparisons between groups were analyzed using a two-tailed Student’s t-test, one-way analysis of variance (ANOVA) followed by Tukey’s or Bonferroni correction for continuous data, Mann-Whitney U test for ordinal data or two-group comparisons where appropriate, or two-way ANOVA followed by a Tukey’s multiple comparison test for the time points and EAE cumulative disease scores where appropriate. Differences were considered statistically significant at *P* < 0.05.

## 3 Results

### 3.1 Expression and cellular distribution of TLR4 in EAE tissues

HMGB1 exhibits immunomodulatory properties when it binds to receptors such as TLR2, TLR4 and RAGE. To investigate the role of HMGB1 and TLR4 in EAE, we examined their expression in the spinal cords of EAE mice. Immunohistochemical staining revealed low levels of HMGB1 and TLR4 expression in naive mice, which increased in the lateral and dorsal columns, as well as the ventral and dorsal horns of EAE mice (**Figure 1A**). Quantitative analysis of HMGB1^+^ and TLR4^+^ cells in total sections, as well as in the white matter (WM) and gray matter (GM) showed that the number of these cells increased in EAE mice (**Figure 1B**). Furthermore, we analyzed the linear regression between TLR4 and HMGB1. As expected, a positive correlation was observed between TLR4 and HMGB1 in the spinal cord, similar to the results seen in the WM and GM of the spinal cord (**Figure 1C**). To identify determine whether TLR4 is expressed in HMGB1, we performed double-fluorescence staining using antibodies for both proteins. Co-localization of HMGB1 with TLR4 was then examined in the WM of the spinal cord. Double fluorescence staining showed that HMGB1/TLR4 colocalized (**Figures 1D**, arrows). In the naive groups, HMGB1 was clearly expressed in the nuclei of cells in the WM of the spinal cord. In particular, HMGB1 expressed in the in the cytoplasm at peak stage in this region.

**Figure 1:**
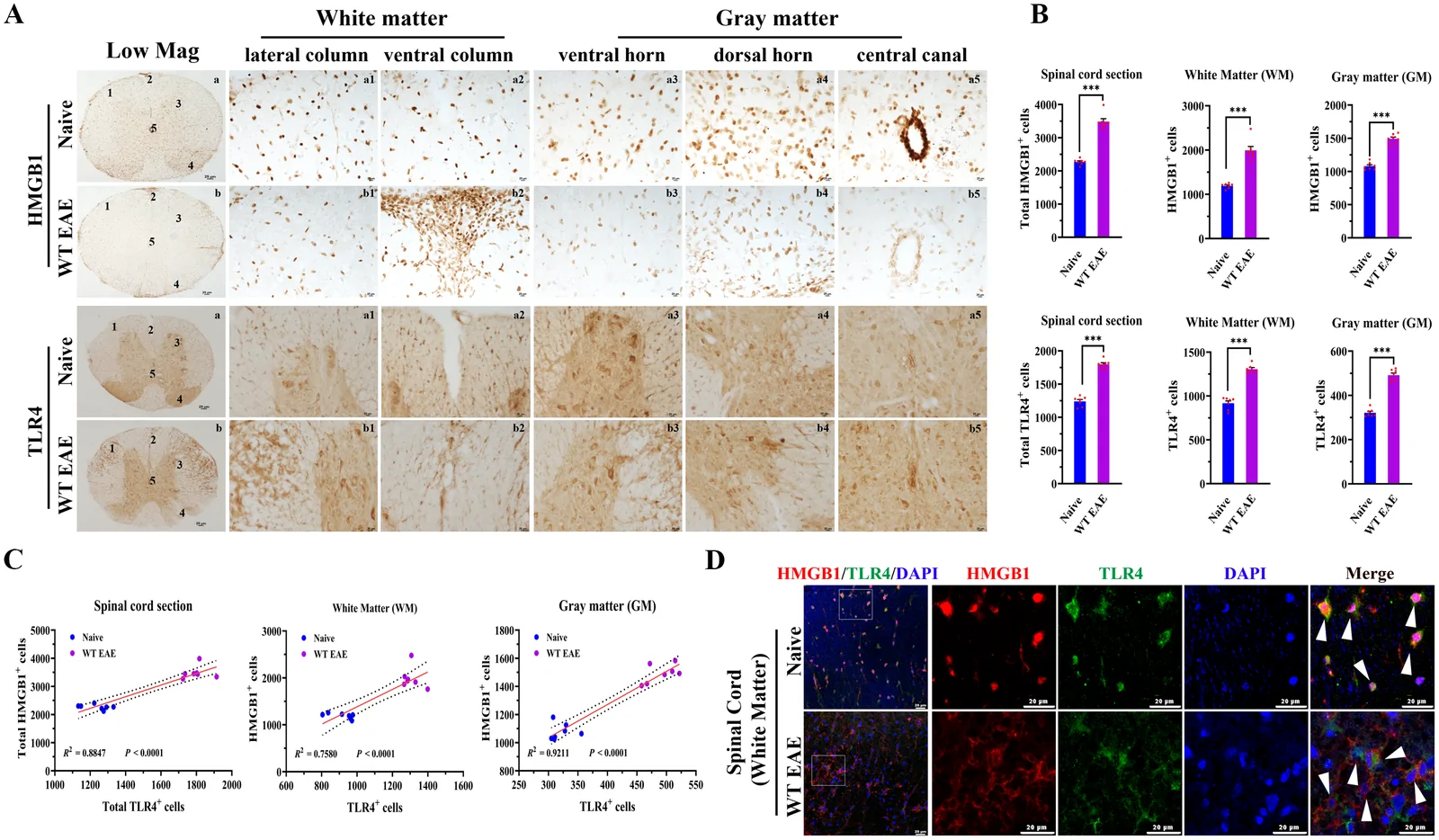
Alteration of TLR4 and HMGB1 expression in spinal cord tissues from EAE mice. **(A)** Representative immunohistochemical (IHC) staining of HMGB1 and TLR4 in thoracic spinal cord sections at the peak stage of EAE. 1: lateral column; 2: dorsal column; 3: ventral horn; 4: dorsal horn. Scale bars: 25 µm (low magnification) and 25 µm (high magnification). **(B)** The number of HMGB1- and TLR4-positive cells in one spinal cord section. The naive group comprises normal mice (n = 7), and the WT EAE group comprises wild-type mice that were induced with EAE (n = 7). Data are expressed as the mean ± SEM. ***P < 0.001 vs naive group, two-tailed Student’s t-test. **(C)** TLR4 expression was positively correlated with HMGB1 expression in spinal cord tissue from mice in the peak EAE phase. **(D)** Representative images of double immunofluorescence staining showing the co-localization (arrows) of HMGB1 (red) and TLR4 (green) in the white matter of the spinal cord (arrows). The cells indicated by the arrows are HMGB1^+^TLR4^+^. Cell nuclei were counterstained with DAPI (blue). Scale bars, 20 μm.

To clarify the role of the HMGB1/TLR4 axis in the CNS, it is essential to identify which CNS-resident cells express the HMGB1 receptor, TLR4. Immunofluorescence analysis of naïve spinal cord white matter (WM) revealed that TLR4 is expressed on multiple cell types, including microglia (**Figure 2A**, *upper panel*), astrocytes (**Figure 2A**, *lower panel*), and neurons. In mice with EAE at the peak of disease, double labeling showed that TLR4 was predominantly expressed in Iba1-positive microglia and GFAP-positive astrocytes. Notably, merged images of the gray matter (GM) demonstrated that TLR4-positive neurons were present in naive mice, but TLR4 staining was largely diminished in NeuN-positive neurons in EAE mice (**Figure 2B**). These findings suggest that under neuroinflammatory conditions, TLR4 expression is upregulated in glial cells while being downregulated in neurons, implying a cell-type-specific shift in the HMGB1/TLR4 signaling axis during CNS pathology.

**Figure 2:**
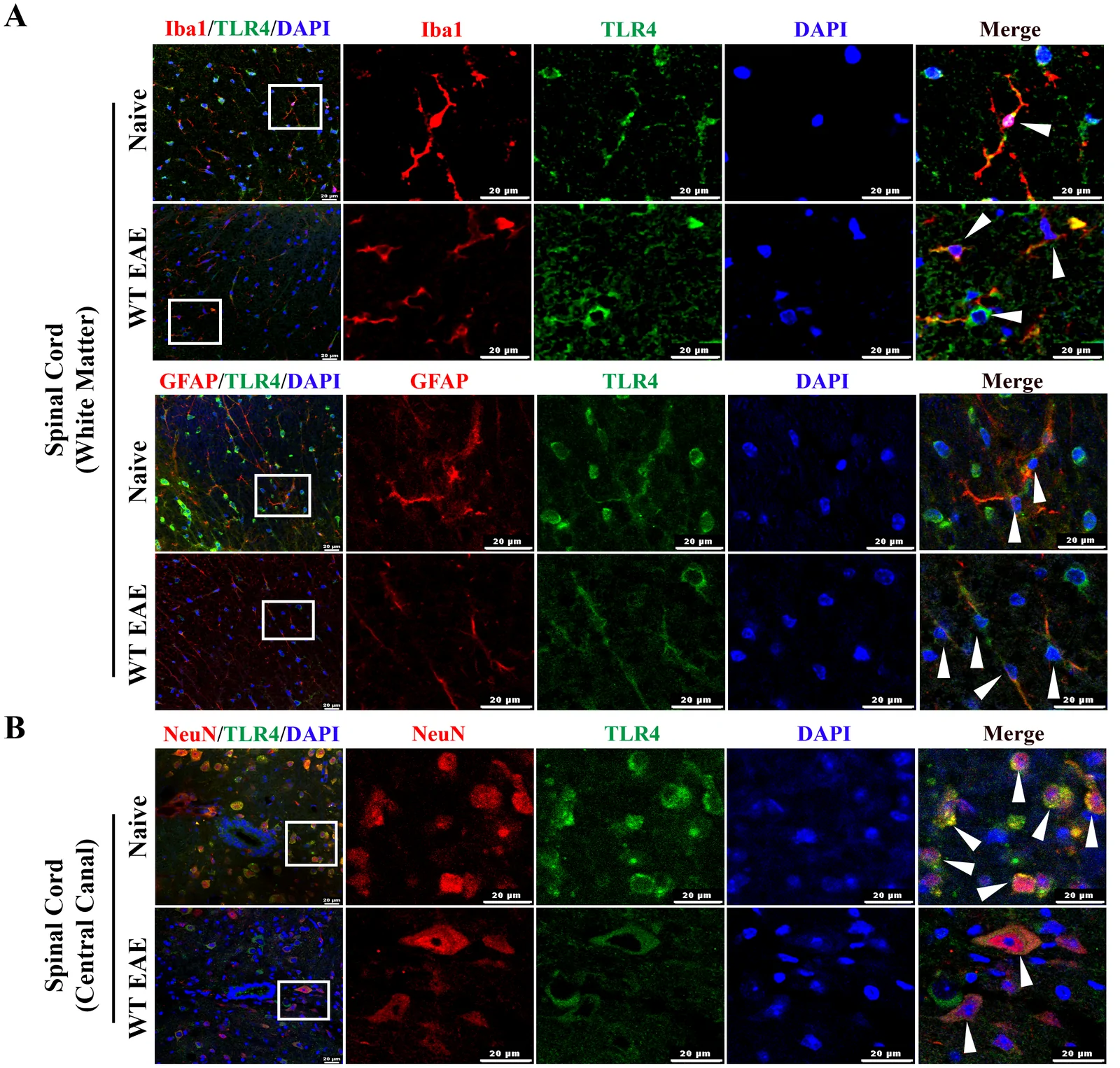
Expression of TLR4 in microglia, astrocytes and neurons in the white matter (WM) and central canal of the naive and EAE spinal cord. **(A)** Representative images of double immunofluorescence staining showing the co-localization (arrows) of TLR4 (green) with the microglia marker Iba1 (red, *upper panel*) and the astrocytes marker GFAP (red, *lower panel*) in the WM. The cells indicated by the arrows are Iba1^+^ TLR4^+^ cells or GFAP^+^TLR4^+^. **(B)** Representative images of double immunofluorescence staining showing co-localization (arrows) of TLR4 (green) with the neuronal markers NeuN (red) in the central canal region. Cells indicated by arrows are NeuN^+^ TLR4^+^. Cell nuclei were counterstained with DAPI (blue). Scale bar, 20 µm.

### 3.2 Decreased EAE severity in the TLR4-deficient mice

The above results suggest that TLR4 activation occurs in EAE tissue. At postnatal weeks 7 - 8, brain sections were prepared from *TLR4*^-/-^ mice, as well as from WT control littermates, for immunofluorescence staining. Compared to the WT group, the *TLR4*^-/-^ group did not express TLR4 globally in the medial prefrontal cortex (mPFC) of the brain or the central canal of the spinal cord (**Figure 3A**). We therefore investigated its role in EAE progression by examining female C57BL/6J WT and *TLR4*^-/-^ mice injected with the MOG_35–55_ peptide. The results showed that WT mice developed severe clinical symptoms characterized by a gradual increase in paralysis severity (**Figure 3B**), whereas *TLR4^-^/^-^* mice exhibited a significantly lower incidence of EAE (78% *vs.* 100%). Even when EAE developed, it was milder, with the mean maximal EAE score being approximately threefold lower (1.08 ± 0.23 *vs.* 3.08 ± 0.11, ***P < 0.001). In addition, the onset of EAE was significantly delayed in *TLR4^-^/^-^* mice (13.64 ± 0.93 days) compared with WT mice (11.00 ± 0.14 days, **P < 0.01, see **Table 1**). *TLR4*^-^/^-^ also decreased the demyelination score, a characteristic pathological manifestation of EAE, as demonstrated by LFB staining (**Figure 3C**). To investigate the impact of TLR4 knockout on inflammatory cell infiltration in the CNS during EAE, a histopathological examination of the spinal cord sections collected at the peak stage of EAE was performed to determine the number of inflammatory cells present. Many mononuclear cells infiltrated the spinal cord parenchyma during EAE. *TLR4*^-/-^ EAE remarkably reduced the immune cell infiltration in the parenchyma (**Figure 3D**, left panel), particularly CD3^+^ T lymphocytes (**Figure 3D**, right panel). Inflammation-related cytokines play a contributory role in EAE pathogenesis and were secreted at significant levels during EAE progression. Here, we found that the levels of TNF-α, IFN-γ and IL-6 declined in both serum and spinal cord homogenate in *TLR4*^-/-^ at peak stage of EAE. In addition, IL-17A was measured in both serum and spinal cord homogenate, and no significant differences were observed between WT and *TLR4*^-/-^ mice at the peak stage of EAE (**Figure 3E**). These results suggest that TLR4 knockout delays the onset of EAE, suppresses its development and reduces its clinical and pathological severity, as well as further neuroinflammation.

**Figure 3:**
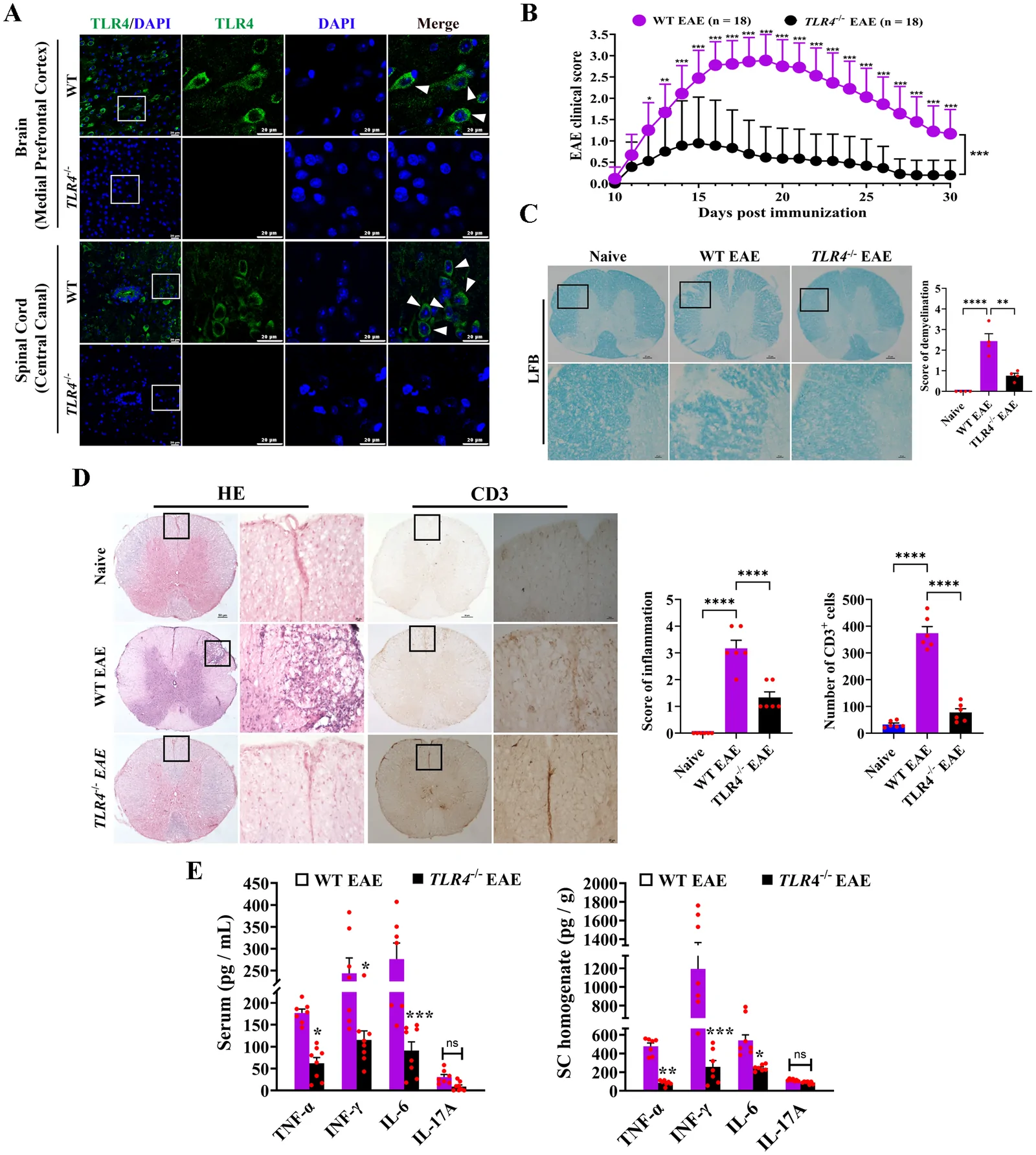
TLR4 deficiency ameliorates EAE via inhibiting CNS inflammation. **(A)** Immunofluorescence detection of TLR4 (green) expressed in the medial prefrontal cortex (mPFC) and central canal of frozen brain sections from WT and *TLR4*^-/-^mice. Nuclei are counterstained with DAPI (blue). Scale bars, 20 μm. mPFC medial prefrontal cortex. **(B)** EAE clinical score of WT and *TLR4^-^/^-^* mice. C57BL/6J mice were immunized with the MOG_35–55_ peptide in CFA. Data are representative of three independent experiments and are shown as median with interquartile range (n = 18/group). *P < 0.05, **P < 0.01, ***P < 0.001 vs. the WT EAE group; Mann-Whitney U test. **(C)** Luxol Fast Blue (LFB) staining to examine demyelination in spinal cord sections from Naive, WT EAE and *TLR4^-^/^-^* EAE mice. Data are representative of three independent experiments and are shown as the mean ± SEM (n = 4/group). **P < 0.01, ****P < 0.0001 vs. the WT EAE group; one-way ANOVA followed by Tukey’s test. **(D)** Hematoxylin (HE) staining to examine the inflammation in spinal cord sections. Immunohistochemistry for CD3. Spinal cord tissues from WT and *TLR4*^−/−^ mice was collected at the peak of EAE after immunization, fixed, sectioned, and stained with HE and CD3, respectively (images are representative of six mice in each group). Scale bars were 50 µm for low magnification and 25 µm for high magnification. Data are shown as the mean ± SEM (n = 6/group; ****P < 0.0001, one-way ANOVA followed by Tukey’s test). **(E)** The concentrations of TNF-α, IFN-γ, IL-6 and IL-17A both in serum and spinal cord homogenate were measured by ELISA at peak stage of EAE. Data are shown as the mean ± SEM, n = 7 - 8 for serum and n = 7 for spinal cord homogenate in each experiment. Data are shown as the mean ± SEM. *P < 0.05, **P < 0.01, ***P < 0.001, two-tailed Student’s t-test.

**Table 1:** Clinical features of EAE in *TLR4*^-/-^ mice.

Group	No. with EAE / total	Incidence (%)	Mean maximal score (day, mean ± SEM)	Disease onset (day, mean ± SEM)
WT EAE	18/18	100	3.08 ± 0.11	11.00 ± 0.14
*TLR4*-/- EAE	14/18	78	1.08 ± 0.23***	13.64 ± 0.93**

Data are representative of three independent experiments (n = 18/group; **P < 0.01, ***P < 0.001, *TLR4*^-/-^ EAE *vs.* WT EAE, using the Mann-Whitney U test).

### 3.3 TLR4 deficiency suppresses the activation of microglia and astrocytes, as well as the damage to neurons caused by EAE

Microglia and astrocytes are resident cells of the CNS. These cells have been reported to participate in the inflammatory process that leads to the onset of EAE. To investigate the effect of TLR4 on inflammatory processes in EAE, we analyzed the activation of microglia and astrocytes in spinal cord sections from *TLR4*^-/-^ mice on day 19, which is during the peak stage of EAE. *TLR4*^-/-^ EAE suppressed the activation of microglia and astrocytes in EAE mice. In the naive group, immunohistochemical staining revealed that slightly staining of Iba1 and GFAP, with the microglia were in a resting state exhibiting thin cell bodies and slender processes. Iba1^+^ (markers for microglia) and GFAP^+^ cells (markers for astrocytes) had small cell bodies and long, thin ramified processes (**Figures 4A, B**, left panels). In the WT EAE group, the microglia and astrocytes were activated and more Iba1 and GFAP were observed. The morphology of the microglia shifted towards enlarged cell bodies with ramified morphology and short, thick processes (**Figures 4A, B**, middle panels). In the *TLR4*^-/-^ EAE group, the number of Iba1^+^ and GFAP^+^ cells were significantly lower than in the WT EAE group (**Figures 4A, B**, right panels). To analyses the effect of TLR4 deficiency on the number of microglia and astrocytes, we quantitatively analyzed the number of Iba1^+^ and GFAP^+^ cells in total sections, WM, or GM. There was a statistically significant difference in the number of Iba1^+^ and GFAP^+^ cells between WT and *TLR4*^-/-^ mice with EAE (**Figure 4C**).

**Figure 4:**
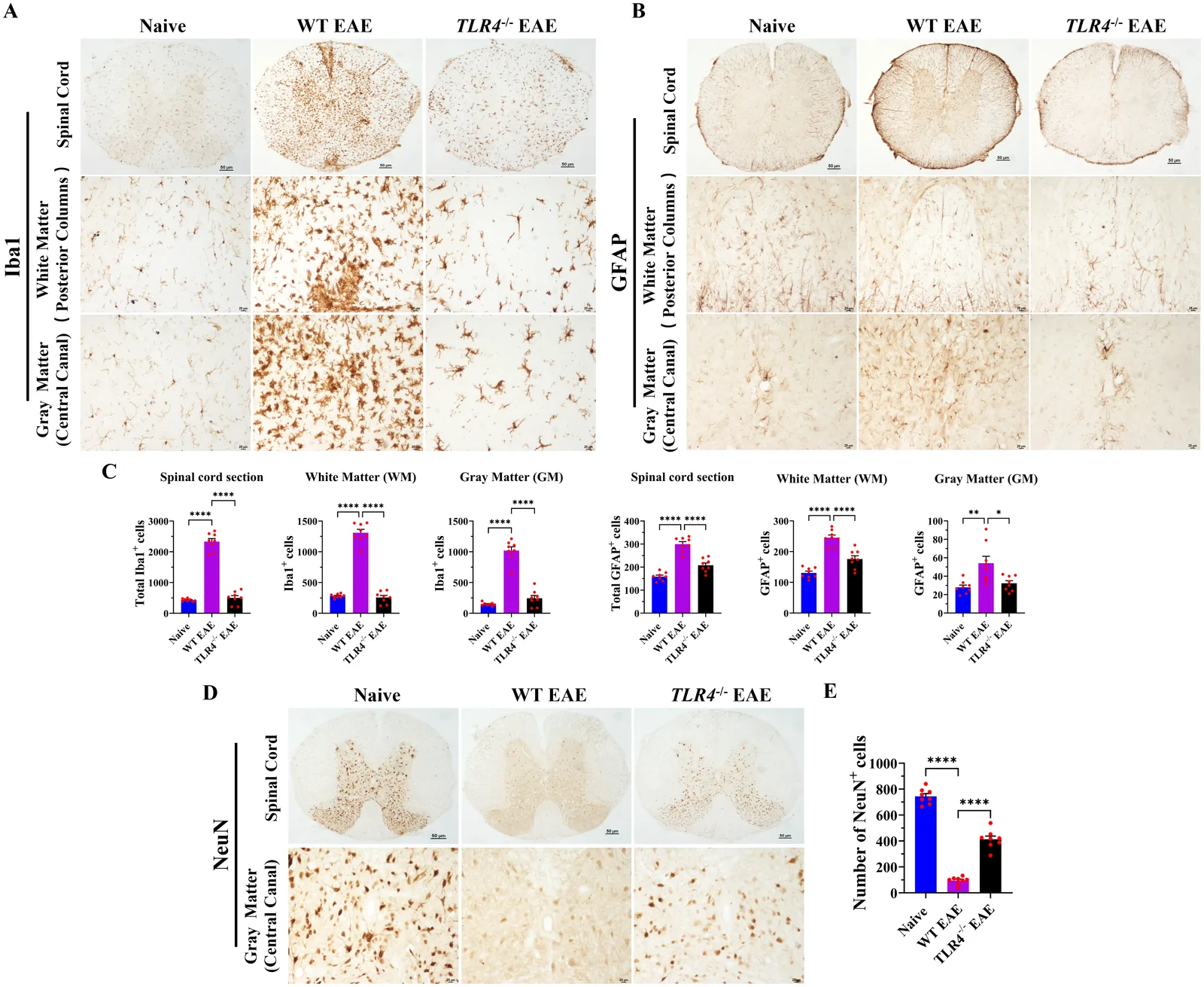
Activated astrocytes, microglia, and damaged neurons in the spinal cords of *TLR4*^-/-^ mice with EAE. Immunohistochemical staining of glial fibrillary acidic protein (GFAP), ionized calcium-binding adapter molecule 1 (Iba1), and neuron-specific nuclear protein (NeuN) in sections of the thoracic spinal cord of WT and *TLR4*^-/-^ mice at the peak stage of EAE. **(A)** Iba1-positive cells. Scale bars were 50 µm for low magnification and 25 µm for high magnification. **(B)** GFAP-positive cells. Scale bars were 50 µm for low magnification and 25 µm for high magnification. **(C)** The number of Iba1- and GFAP-positive cells in the spinal cord. Data are shown as the mean ± SEM (n = 8/group; ****P < 0.0001, one-way ANOVA followed by Tukey’s test). **(D)** NeuN-positive cells. Scale bars were 50 µm for low magnification and 25 µm for high magnification. **(E)** The number of NeuN-positive cells in spinal cord. Data are shown as the mean ± SEM (n = 8/group; ****P < 0.0001, one-way ANOVA followed by Tukey’s test).

Next, we investigated the survival of spinal cord neurons in the entire GM region of MOG_35–55_-immunized mice at the peak stage were investigated using immunohistochemical staining for NeuN (a neuronal marker). Damage to neurons was observed as smeared NeuN staining. The number of NeuN^+^ neurons had increased significantly increased in the *TLR4*^-/-^ EAE mice compared to the WT EAE mice. (**Figures 4D, E**). Together, these data suggest that neuronal damage in the spinal cord, as well as activation of microglia (Iba1^+^ cells) and gliosis (GFAP^+^ cells), may be alleviated in TLR4 knockout mice with EAE. Given the well-established role of peripheral Th1 and Th17 cells in driving neuroinflammation in MS/EAE, we hypothesized that the protective effects observed within the CNS might be linked to the regulation of these peripheral immune responses by TLR4 deficiency. To test this, we examined MOG-specific T cell responses in peripheral lymphoid organs further.

### 3.4 Reduced IFN-γ and IL-17A production in the spleen and draining lymph node supernatant of TLR4-deficient mice

To investigate the immunological mechanisms associated with the reduced severity of EAE in *TLR4*^-/-^ mice, spleen and draining lymph node (DLN) samples were collected at 19 (when clinical symptoms were at their peak) days after immunization. These samples were then restimulated and cultured in the presence or absence of the MOG_35–55_ peptide for 72 h. The resulting supernatant was then harvested and analyzed by ELISA for TNF-α, IFN-γ and IL-17A. Compared with cells from WT EAE mice, spleen and DLN cells obtained from *TLR4*^-/-^ EAE mice displayed reduced IFN-γ and IL-17A production in response to MOG_35-55_ peptide restimulation (**Figures 5A, B**). Notably, TNF-α was also lower in unstimulated cultures from *TLR4*^-/-^ EAE mice, consistent with an intrinsic effect of TLR4 deficiency on myeloid cells. Taken together, these results suggest that TLR4 deficiency suppresses MOG_35-55_-specific Th1/Th17 effector responses, as evidenced by the reduced IFN-γ and IL-17A production upon antigen restimulation.

**Figure 5:**
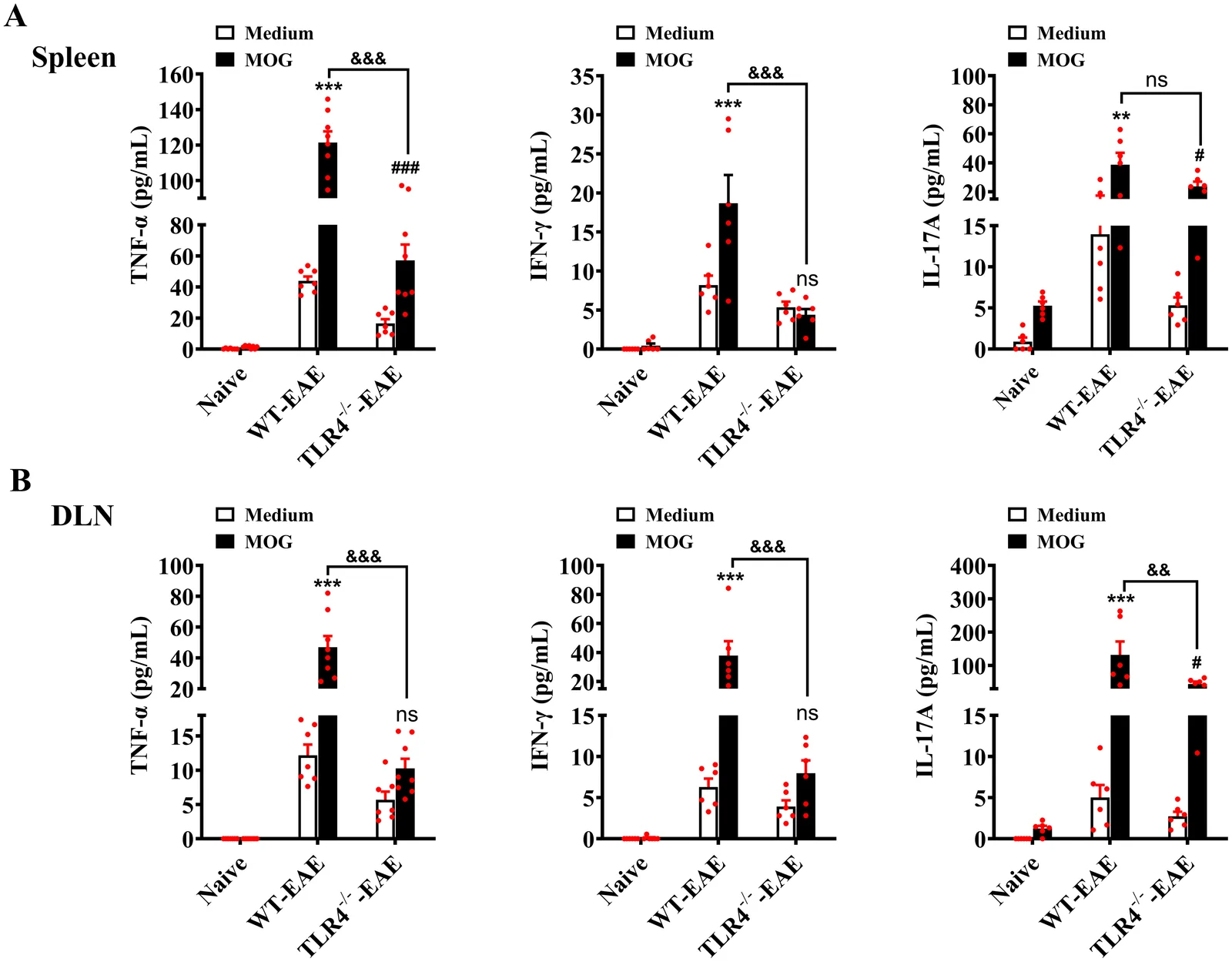
MOG_35–55_ -specific T cell responses in the culture supernatant of spleen and draining lymph node (DLN) cells of *TLR4*^-/-^ mice with EAE. The spleens and DLNs were collected on day 19 during the peak stage of EAE after immunization. The cells were then stimulated with MOG_35–55_ for 72 h. **(A)** The levels of TNF-α, IFN-γ and IL-17A in the supernatant of spleen were determined using ELISA in naive, WT-EAE and *TLR4^-^*^/--^EAE mice. **(B)** The levels of TNF-α, IFN-γ and IL-17A in the supernatant of DLN. Data shown below are pooled from two independent experiments and are shown as the mean ± SEM (n = 6 - 8/group; **P < 0.01, ***P < 0.001, ^&&^*P* < 0.01, ^&&&^*P* < 0.001, ^#^*P* < 0.05, ^###^*P* < 0.001, ns: not significant, one-way ANOVA followed by Tukey’s test).

### 3.5 TLR4 deletion regulated transcription factors related to Th1 and Th17 cells in spinal cord

As MOG-specific Th1/Th17 responses were found to be reduced in peripheral lymphoid organs, we investigated whether TLR4 deficiency also affects Th1/Th17-related transcription factors in the CNS. We examined the mRNA expression of TNF-α, IFN-γ and IL-17A, as well as the related Th1 and Th17 transcription factors T-bet and RORγt in the spinal cord. The expression of mRNA for TNF-α, IFN-γ, IL-17A, T-bet and RORγt increased on day 19 compared to the control group, revealing that EAE may induce an immune shift towards Th1 and Th17 cell subsets in the CNS. Compared to the WT-EAE group, *TLR4*^-/--^EAE reversed the elevated levels of TNF-α, IFN-γ, IL-17A, T-bet and RORγt mRNA in the spinal cord at the peak EAE stage (**Figure 6**). These results suggest that TLR4 deficient mice may suppress the differentiation of Th1 and Th17 cells in the spinal cord after EAE by regulating the related transcription factors T-bet and RORγt.

**Figure 6:**
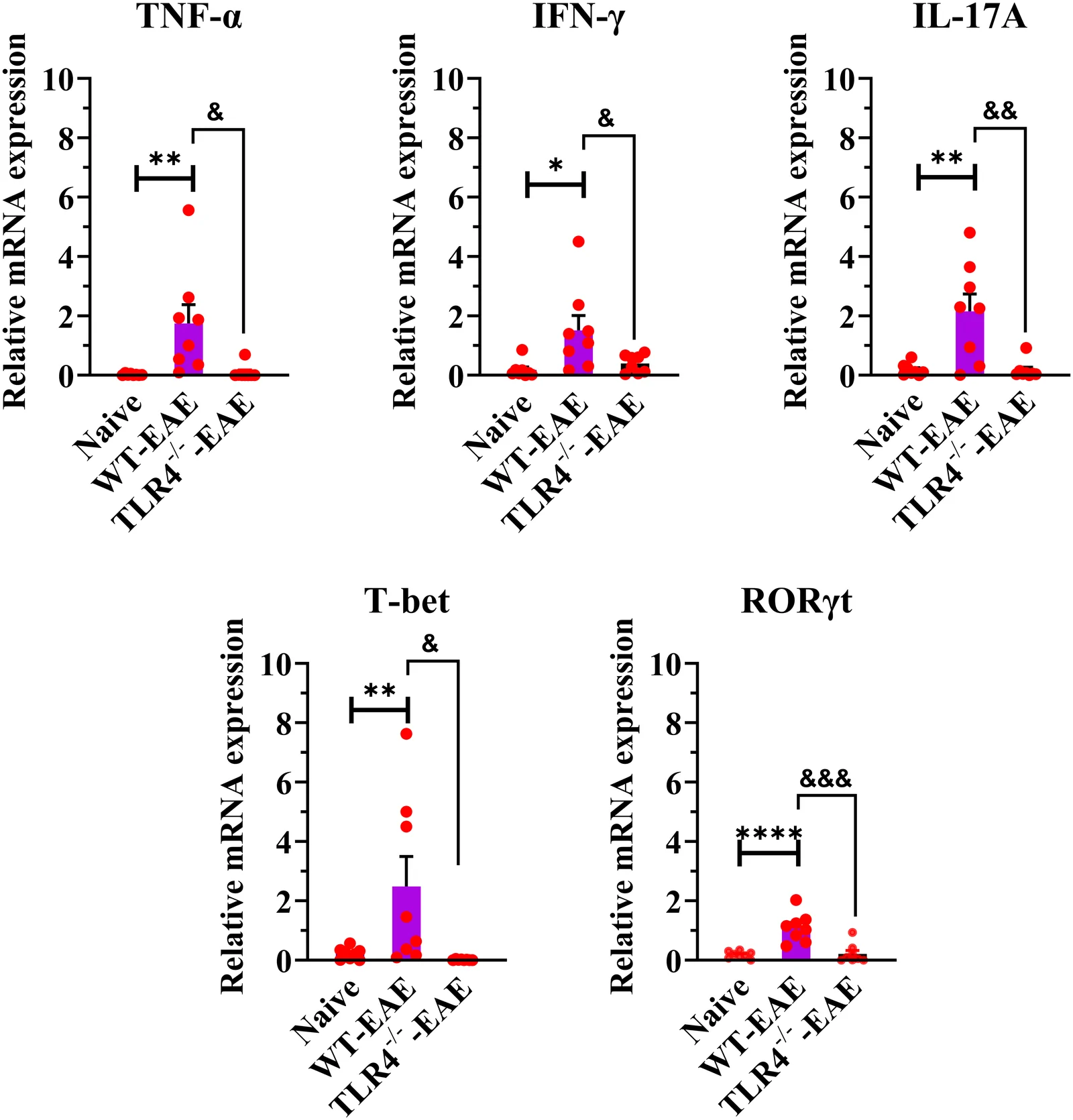
Expression of TNF-α, IFN-γ, IL-17A, T-bet and RORγt mRNA in the spinal cords of MOG_35–55_-immunized WT and *TLR4*^-/-^ mice during the peak stage of EAE. Spinal cord tissues were harvested on day 19 to measurement TNF-α, IFN-γ and IL-17A mRNA using quantitative PCR (qPCR). qPCR analysis was performed to determine the relative mRNA expression levels of T-bet and RORγt in spinal cord tissues. Data were normalized to the housekeeping gene GAPDH, as described in the Materials and Methods section. Data shown below are pooled from two independent experiments and are shown as the mean ± SEM (n = 8/group; *P < 0.05, **P < 0.01, ****P < 0.0001, ^&^*P* < 0.05, ^&&^*P* < 0.01, ^&&&^*P* < 0.001, one-way ANOVA followed by Tukey’s test).

### 3.6 TAK-242 alleviates symptoms and delays the onset of EAE in mice

To investigate the impact of TAK-242 on the initiation and progression of EAE, mice were treated using three distinct strategies, as illustrated in **Figure 7A**. Similar treatment strategies have been reported by our research group and others (20, 30). In Strategy I, injection was given from days -1 to 29 (an early prophylactic TAK-242 treatment regimen); in Strategy II, from days 11 to 21 (a mid-to-late TAK-242 treatment regimen); and in Strategy III, from days 15 to 23 (a late TAK-242 treatment regimen). Early prophylactic TAK-242 treatment significantly influenced EAE incidence, mean maximal score, disease onset and long-term protective effects in EAE mice (**Figure 7B**; **Table 2**). Midterm and late treatment with TAK-242 significantly attenuated disease severity (**Figures 7C, D**; **Table 2**). As shown in **Table 2**, mid-to-late treatment with TAK-242 clearly decreased the disease incidence. These data indicate that TAK-242 treatment confers the prophylactic and therapeutic effects in EAE mice.

**Figure 7:**
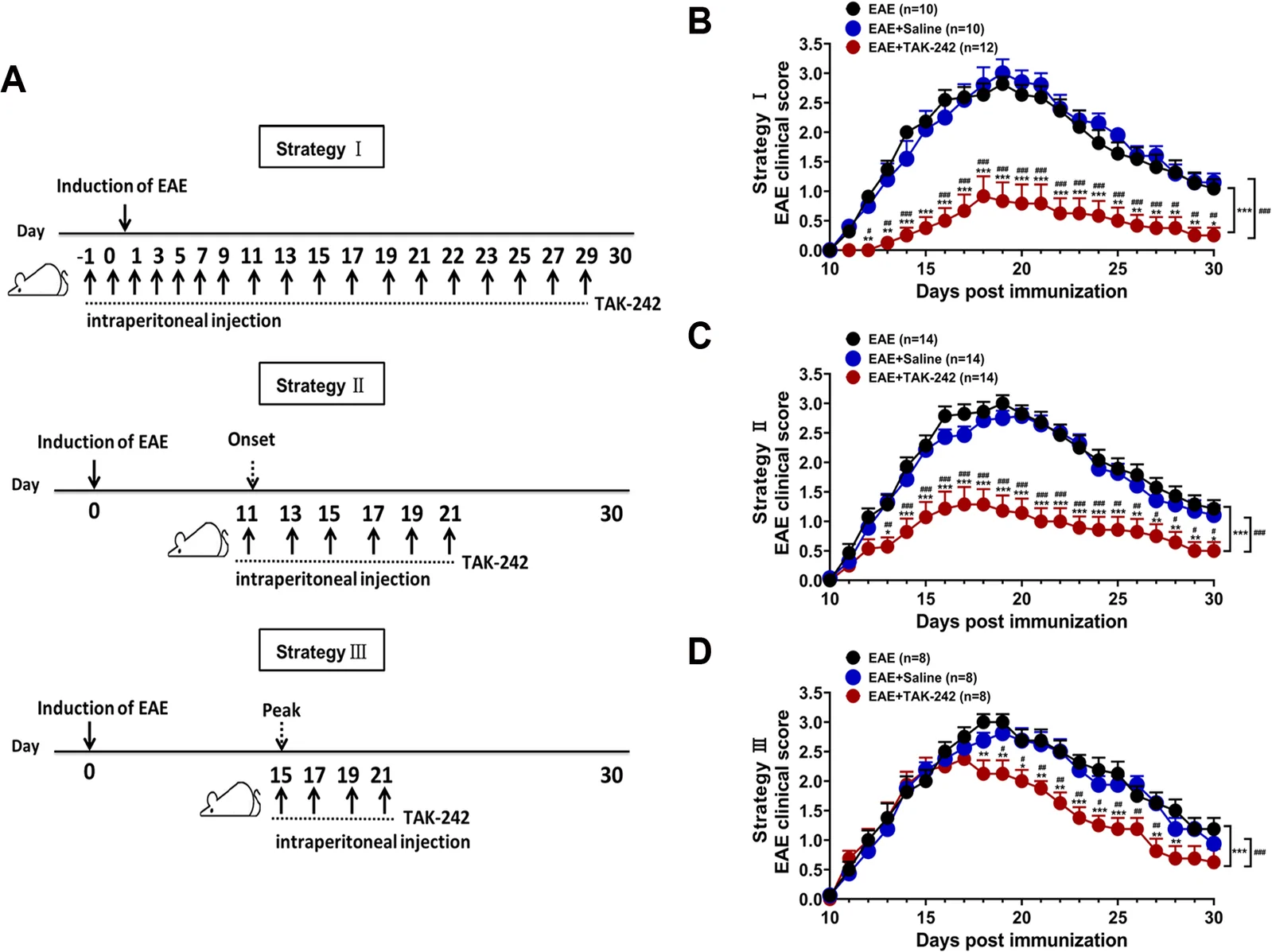
The effects of TAK-242 on the development of EAE. **(A)** Schematic workflow of TAK-242 treatment. Mice were injected intraperitoneally (i.p.) with either 25 mg/kg of TAK-242 or 0.9% saline solution each time. **(B–D)** The effects of TAK-242 treatment on disease score. In Strategy I, mice were injected i.p. from days -1 to 29; in Strategy II, from days 11 to 21 and in Strategy III, from days 15 to 23, with TAK-242 or saline solution. *P < 0.05, **P < 0.01, ***P < 0.001 EAE + TAK-242 *vs.* EAE group; ^#^*P* < 0.05, ^##^*P* < 0.01, ^###^*P* < 0.001 EAE + TAK-242 *vs.* EAE + saline group. A two-way ANOVA followed by a Tukey’s multiple comparison test was used to analyze the time points or cumulative disease scores. The results shown represent one or two independent experiments.

**Table 2:** Clinical features of EAE mice with TAK-242 treatment.

Strategy I : pre-onset to chronic stage					Strategy II : onset and peak stages				Strategy Ill: peak stage			
Group	No.with EAE/total	Incidence (%)	Mean maximal score (mean ±SEM)	Disease onsett (day,mean ±SEM)	No.with EAE/total	Incidence (%)	Mean maximal score (mean ±SEM)	Disease onset (day, mean ±SEM)	No.with EAE/total	Incidence (%)	Mean maximal score (mean ±SEM)	Disease onset (day, mean ±SEM)
EAE	10/10	100	2.95 ± 0.16	11.50 ± 0.17	14/14	100	3.07 ± 0.15	11.43 ± 0.14	8/8	100	3.06 ± 0.15	11.13 ± 0.23
EAE + Saline	10/10	100	3.00 ± 0.24	11.40 ± 0.22	14/14	100	3.00 ± 0.09	11.64 ± 0.23	8/8	100	2.88 ± 0.16	11.25 ± 0.31
EAE + TAK-242	7/12	59	1.57 ± 0.43***,###	15.00 ± 0.70***,###	12/14	86	1.63 ± 0.26***,###	12.83 ± 0.72*,#	8/8	100	2.38 ± 0.16**,#	11.38 ± 0.38

“EAE”: EAE induction without treatment. “EAE + Saline”: EAE induction followed by treatment with a 0.9% saline solution. “EAE + TAK-242”: EAE induction with treatment of 25 mg/kg TAK-242. **P < 0.01, ***P < 0.001, *vs.* the EAE group; ^#^*P* < 0.05, ^###^*P* < 0.001, *vs.* the EAE + Saline group, using the Mann–Whitney U test. The results shown are representative of two independent experiments.

## 4 Discussion

The core finding of this study is that TLR4 plays a pivotal role in the development of EAE. We demonstrated that activation of the TLR4 signaling pathway promotes neuroinflammation, demyelination and neuronal damage in EAE. Conversely, inhibiting this pathway (through either gene knockout or pharmacological blockade) significantly alleviates disease severity. The following discussion integrates our findings with existing literature to explore the mechanisms of TLR4 in MS/EAE and its therapeutic potential.

Our immunohistochemical analysis revealed significant upregulation of TLR4 expression in the spinal cords of EAE mice, particularly in white matter regions. This finding is consistent with previous studies indicating that the neuroinflammatory environment can induce TLR4 expression (16). More importantly, the change in its cellular distribution is noteworthy. In naive mice, co-localization of TLR4 with the neuronal marker NeuN was observed, suggesting that basal neuronal TLR4 expression may be involved in physiological functions or low-level surveillance. However, during the peak stage of EAE, this TLR4/NeuN co-localization decreased, possibly reflecting severe neuronal damage or apoptosis. Concurrently, TLR4 expression shifted predominantly to GFAP-positive astrocytes and Iba1-positive microglia. This shift in distribution is crucial.

The apparent downregulation of TLR4 in NeuN^+^ neurons during EAE may reflect several non-mutually exclusive mechanisms. Firstly, sustained exposure to endogenous TLR4 ligands, such as HMGB1 and HSPs, which are released during neuroinflammation may promote receptor internalization and degradation, reducing detectable neuronal TLR4 despite upregulation in glial cells. Secondly, downregulation of neuronal TLR4 may represent a cell-intrinsic homeostatic adaptation within the cells themselves that limits excessive NF-κB-mediated neurotoxicity in cells with limited regenerative capacity. Thirdly, the regulation of TLR4 may be temporally dynamic, involving transient early upregulation followed by downregulation at peak stages, whereas glial upregulation persists. Fourthly, some of the apparent reduction may result from the loss or damage of NeuN^+^ neurons during EAE, as has been observed in MS/EAE tissues. Further investigation is required to determine whether these mechanisms are operating in parallel or independently, including systematic time-course analysis and cell-type-specific manipulation.

The activation of microglia and astrocytes in the CNS via TLR4 plays a key role in driving neuroinflammation. TLR4 recognizes various endogenous DAMPs, such as HMGB1, which is mentioned in the background to this study. In MS/EAE, myelin destruction and neuronal damage release large quantities of DAMPs, such as HMGB1 (17). Our results suggest that these DAMPs may initiate pro-inflammatory signaling cascades downstream by activating TLR4 on glial cells. This view is supported by the literature, the HMGB1-TLR4 axis has been shown to activate microglia, leading to the release of cytokines such as TNF-α and IL-6. These cytokines then disrupt the BBB and recruit peripheral immune cells (18). Therefore, the upregulation of TLR4 expression and its redistribution to glial cells in EAE tissues marks the transition of TLR4 from a surveillance receptor in a resting state to an ‘active participant’ that drives pathological neuroinflammation.

TLR4 knockout (*TLR4*^-/-^) mice exhibited a reduced incidence of EAE, a delayed onset and significantly lower clinical and pathological scores (inflammation and demyelination). This directly confirms the necessity of TLR4 signaling in EAE pathogenesis. Our mechanistic exploration reveals the protective effects of TLR4 deletion from multiple angles.

A key hallmark of CNS inflammation is the morphological shift of microglia from a resting state (thin cell bodies with slender processes) to an activated state (enlarged cell bodies with short, thick processes). We found that *TLR4*^-/-^ significantly suppressed the pathological activation of microglia induced by EAE. This aligns with the reduced levels of pro-inflammatory factors (TNF-α, IFN-γ and IL-6) in serum and spinal cord homogenates. The classic TLR4 downstream pathway is the MyD88-dependent pathway, which ultimately activates NF-κB and MAPKs, driving the transcription of pro-inflammatory genes (19). TLR4 deletion likely interrupts this pathway, thereby curbing the overactivation of microglia and astrocytes, as well as the cytokine storm at its source.

Long-term disability in EAE is closely associated with irreversible neuronal damage and loss. Our study showed that, compared to WT EAE mice, *TLR4*^-/-^ EAE mice had significantly more surviving NeuN^+^ neurons in the grey matter. This is likely to be a direct consequence of the anti-inflammatory effects. The attenuated inflammatory environment (e.g. reduced TNF-α and IL-6 levels) decreases excitotoxicity, oxidative stress and apoptotic signaling attacks on neurons (20). Furthermore, some studies suggest that TLR4 signaling itself may directly contribute to the induction of neuronal apoptosis pathways (21). Therefore, TLR4 deletion may protect neurons through direct and indirect mechanisms.

EAE, particularly the MOG_35-55_-induced model in C57BL/6J mice, is primarily mediated by CD4^+^ T cells, particularly the Th1 and Th17 subsets (22). Our data showed that cells from the spleen and draining lymph nodes of *TLR4*^-/-^ mice produced lower levels of IFN-γ (a Th1 signature cytokine) and IL-17A (a Th17 signature cytokine) upon MOG_35-55_ restimulation. It should be noted that TNF-α is produced not only by T cells, but also by antigen-presenting cells, such as macrophages, dendritic cells and other myeloid cells. In our study, TNF-α levels were significantly reduced in both MOG_35-55_-restimulated and unstimulated cultures from *TLR4*^-/-^ mice, suggesting that the observed decrease may partly result from an intrinsic effect of TLR4 deficiency on innate immune cells, rather than being solely a result of a reduction in antigen-specific T cell responses. Therefore, the TNF-α data should be interpreted with caution, and additional assays (e.g. intracellular cytokine staining or cell-specific knockout models) are required to identify the cellular sources of TNF-α in this context. Furthermore, at the level of spinal cord tissue, TLR4 deletion reversed the upregulation of mRNA for the key Th1 transcription factors T-bet and RORγt induced by EAE. These findings reveal a deeper mechanism by which TLR4 regulates EAE, influencing the differentiation and effector functions of T cells in both the periphery and the CNS. The mechanism may involve several aspects: Firstly, TLR4-activated antigen-presenting cells (e.g. microglia and dendritic cells) secrete cytokines such as IL-6 and IL-23, which are crucial for Th17 differentiation and maintenance (23). Secondly, TLR4 signaling may influence the expression of co-stimulatory molecules, thereby optimizing conditions for T cell activation. Thirdly, Th1/Th17 cells that infiltrate the CNS can be further sustained and expanded by cytokines (e.g. TNF-α, L-6 and IL-1β) produced by locally TLR4-activated glial cells, creating a vicious cycle (24). TLR4 deficiency breaks this cycle, leading to a dampened pathogenic T cell response.

Studies on *TLR4*^-/-^ mice provided genetic evidence, while the application of the TLR4-specific inhibitor TAK-242 validated the feasibility of pharmacological intervention. The *TLR4*^-/-^ mouse line used in this study was generated and characterized in foundational studies by Poltorak et al. (14) and Hoshino et al. (15), which established that loss of TLR4 abolishes LPS signaling *in vivo* and validated this line as a faithful loss-of-function model for TLR4. TAK-242 binds to the intracellular domain of TLR4, inhibiting its interaction with downstream adaptor proteins and thus blocking signal transduction (13). We employed three administration strategies, and the results are instructive: Early prophylactic administration (Strategy I) was the most effective approach, reducing the incidence of the condition, delaying its onset and lowering maximum scores, while providing long-term protection. This suggests that blocking TLR4 during the initiation (sensitization) phase of the autoimmune response may affect the initial activation and differentiation of T cells. Mid-to-late (strategy II) and late (strategy III) therapeutic administration also significantly reduced disease severity. This indicates that, even after clinical symptoms appear and inflammation is established, inhibiting TLR4 signaling can effectively control disease progression. These findings provide stronger support for targeting TLR4 as a therapeutic approach for clinical translation, given that most MS patients are already in an active disease phase at the time of diagnosis. Our results are consistent with those of recent studies that have used TAK-242 in EAE models (25). Together, these studies suggest that TLR4 inhibition is a promising therapeutic strategy for neuroimmune diseases.

It should be noted that the role of TLR4 in EAE is a matter of debate. While Monica Marta et al. reported a regulatory role for TLR4/TLR9 in EAE (26), whereas Miranda-Hernandez et al. suggested that EAE is mainly dependent on MyD88/TLR2/TLR9, with TLR4 playing no substantial role (27). However, this is not a consistent finding. As early as 2004, Steven M Kerfoot et al. reported that EAE was less severe in *TLR4*^-/-^ mice than in wild-type (WT) mice (28). These discrepancies may be due to differences in mouse strain, immunization protocol, adjuvant, and disease stage. In our standardized MOG_35–55_ induced C57BL/6J EAE model, both the genetic deletion of TLR4 and the pharmacological inhibition of TLR4 using TAK-242 consistently alleviated disease severity. This supports the idea that TLR4 plays a pro-inflammatory role in this setting.

TLR4 signaling may promote the differentiation, infiltration and effector functions of pathogenic Th1 and Th17 cells in both the periphery and the CNS by modulating antigen-presenting cell function and the inflammatory microenvironment. These activated Th1/Th17 cells then exacerbate CNS inflammation and damage by releasing more DAMPs, thus forming a positive feedback loop that amplifies the disease process. Genetic deletion or pharmacological inhibition of TLR4 interrupts this critical node in the loop by suppressing inflammation from both the innate and adaptive immune systems. This ultimately results in reduced demyelination, protection of neurons, and improved clinical symptoms.

It is important to note that the production of antigen-specific cytokines and cell proliferation are regulated independently. Effector T cells can produce cytokines within hours of antigen encounter without entering the cell cycle. However, sustained TCR stimulation is required for clonal expansion (29, 30). Dissociations between proliferation and cytokine output have been documented in antigen-specific T cell lines and clones (31, 32). In the context of EAE, the pathogenic capacity of Th1 and Th17 cells is determined by their cytokine program rather than the extent of their expansion (33, 34). Therefore, the reduced production of TNF-α, IFN-γ and IL-17A observed in *TLR4*^-/-^ EAE mice upon MOG_35–55_ restimulation reflects a suppressed effector cytokine response, which is a functional readout in its own right.

From a clinical perspective, MS a human disease with pathological similarities to EAE, also shows elevated expression of TLR4 and its ligands HMGB1 in lesion tissues (16, 35). Therefore, targeting the HMGB1- TLR4 axis could provide a new way to treat MS. The safety of TAK-242 has been established in clinical trials for sepsis (36), facilitating its repurposing for the treatment of neuroimmune diseases. Further research is needed to investigate the combined effects of TLR4 inhibitors alongside existing MS disease-modifying therapies (e.g. interferons, fingolimod and CD20 monoclonal antibodies), as well as their efficacy in animal models of various MS subtypes, such as relapsing-remitting and progressive.

This study also has some limitations. Firstly, the global knockout of TLR4 in mice means that the relative contributions of TLR4 to the hematopoietic (immune cell) system versus the non-hematopoietic (central nervous system resident cell) system cannot be distinguished. In particular, the relative contributions of neuronal versus glial TLR4 cannot be distinguished. Future studies using bone marrow chimeras, cell-specific conditional knockout mice, or neuron-specific conditional TLR4 deletion (e.g. crossed *TLR4*^fl/fl^ with a neuronal Cre driver) as well as neuron-targeted viral approaches will be necessary to define the cell-type-specific functions of TLR4 in EAE, and to distinguish between neuronal and glial contributions. Secondly, while our focus was primarily on Th1/Th17 responses, the potential impact of TLR4 on regulatory T cell (Tregs) function and that of other immune cell subsets remains to be explored. Thirdly, only a limited panel of serum cytokines was measured; the absence of serum IL-1β and IL-10, as well as the inability to quantify CNS-localized cytokines, are acknowledged as limitations, and an expanded cytokine panel is listed as a future task. Fourthly, antigen-specific proliferation was not assessed, and that a bulk-culture proliferation readout would not distinguish between T cell-intrinsic and antigen-presenting cell (APC)-mediated effects. As antigen-specific proliferation was not assessed in this study; therefore, our conclusions are restricted to effector cytokine production. In addition, because TNF-α can be produced by both T cells and myeloid cells, the cellular source of the reduced TNF-α in *TLR4*^-/-^ EAE cultures could not be determined in our bulk-culture system. Fifthly, the specific dosage, pharmacokinetics and distribution concentration of TAK-242 in the CNS require further optimization and clarification.

This study provides a multi-level chain of evidence confirming the central role of the TLR4 signaling pathway in initiating, amplifying and mediating neuronal damage in EAE neuroinflammation. Inhibiting TLR4, either by gene knockout or using the small molecule inhibitor TAK-242, effectively reduces disease severity. This involves suppressing the activation of innate immune cells, reducing the production of pro-inflammatory cytokines, attenuating pathogenic Th1/Th17 responses and protecting neuronal survival. These findings deepen our understanding of the molecular mechanisms underlying EAE/MS pathogenesis and provide a solid preclinical basis for developing novel immunomodulatory therapies that target TLR4.

## Data Availability

The original contributions presented in the study are included in the article/supplementary material. Further inquiries can be directed to the corresponding authors.
